# Supplementary figures and images for: Genomic and phenotypic analyses suggest moderate fitness differences among Zika virus lineages
Source: PLoS Negl Trop Dis. 2023 Feb 8;17(2):e0011055. doi: 10.1371/journal.pntd.0011055 (PMC9907835; doi:10.1371/journal.pntd.0011055)

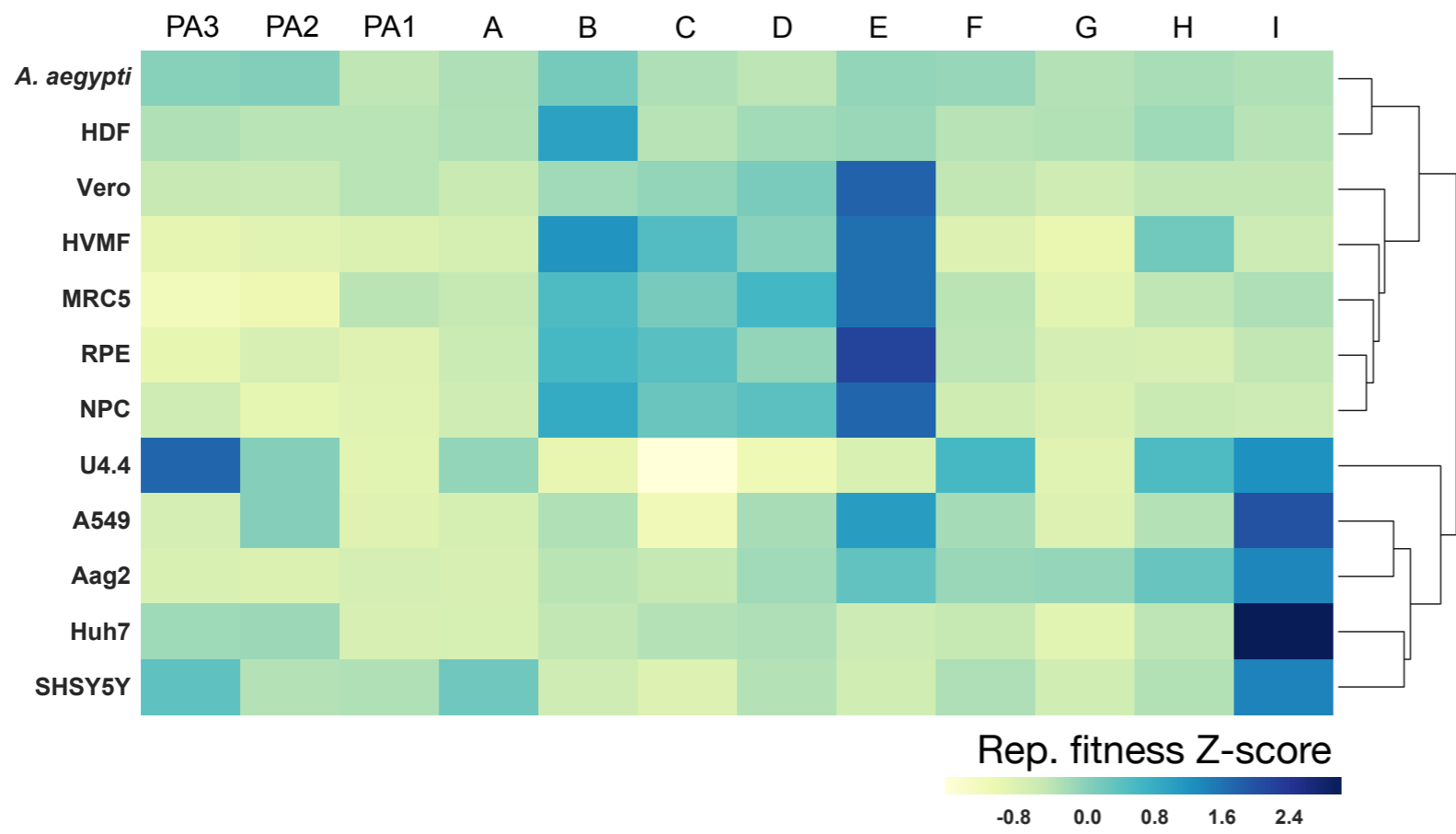

Supplement: S1 Fig — To compare fitness values across cell and mosquito models, we averaged the day three and day four (just day three for A549) replicative fitness values and converted them to Z-scores. Then the Z-scores for each model were clustered (agglomerative hierarchical clustering). Cell types are explained in (S2 Table). (PDF) [file pntd.0011055.s001.pdf]

Vero (MOI .1)

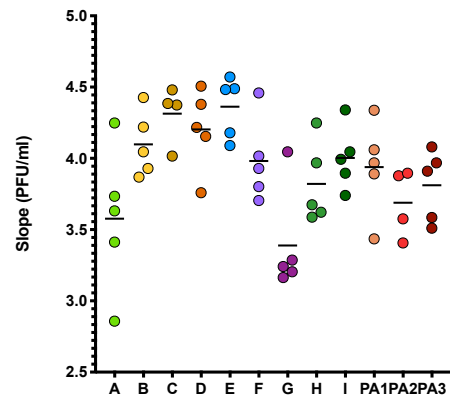

MRC5 (MOI .5)

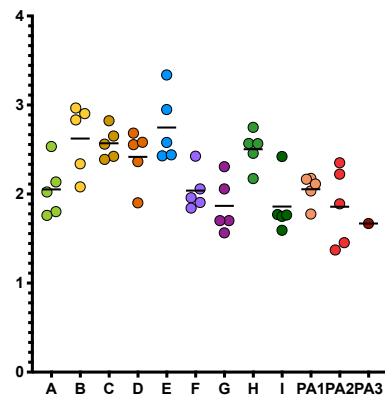

A549 (MOI .1)

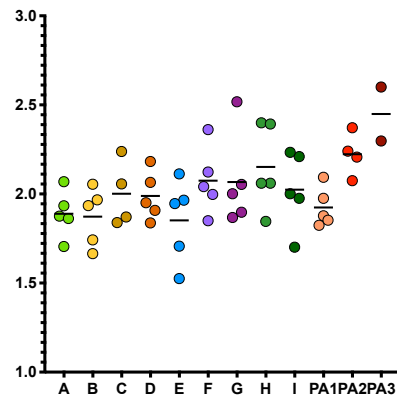

Huh7 (MOI .1)

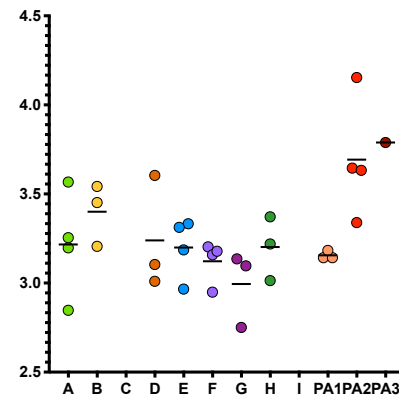

RPE (MOI .5)

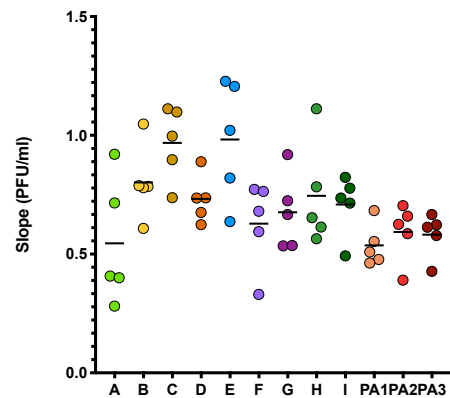

HVMF (MOI .5)

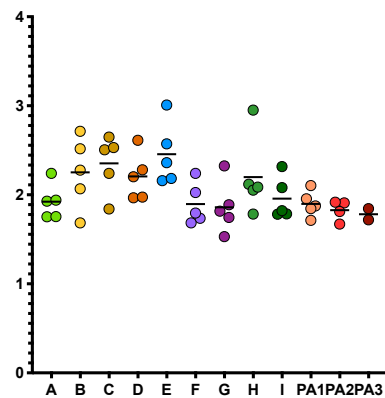

NPC (MOI .5)

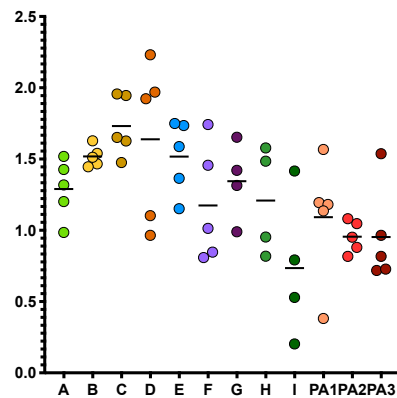

HDF (MOI .5)

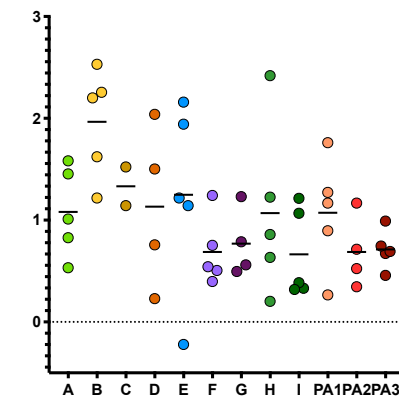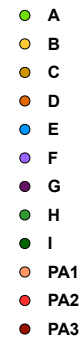

Supplement: S2 Fig — As a secondary endpoint to assess replicative fitness differences, we measured the growth rates of the replicative fitness curves across all 12 clades by fitting a line to every curve for all time points under exponential growth. Using this method, we are able to control for any variation in the infectious dose of the initial inocula. While there were no statistical differences found using this method, clades B, C, D, and E had the highest growth rates among primary human cells. Cell types are explained in (S2 Table). (PDF) [file pntd.0011055.s002.pdf]

**a**

Titers  
(Day 0 - Engorged)

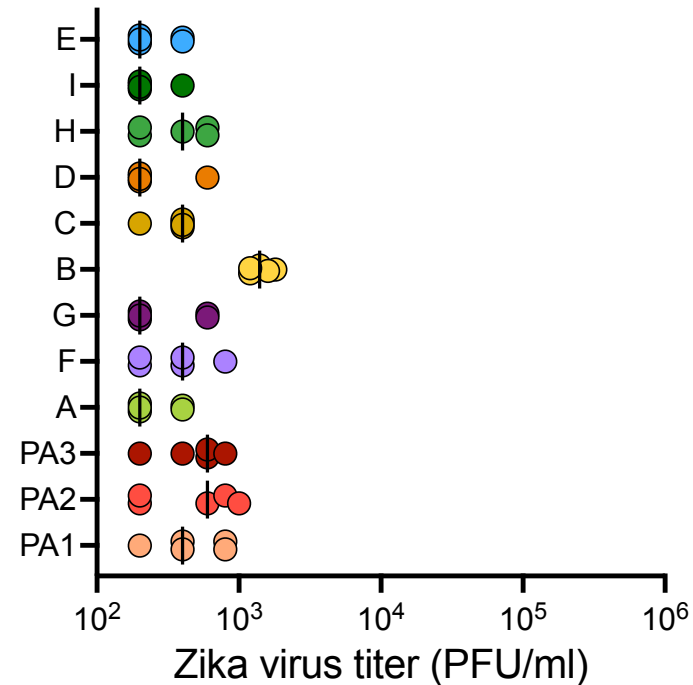**b**

Titers  
(Day 14 - Disseminated)

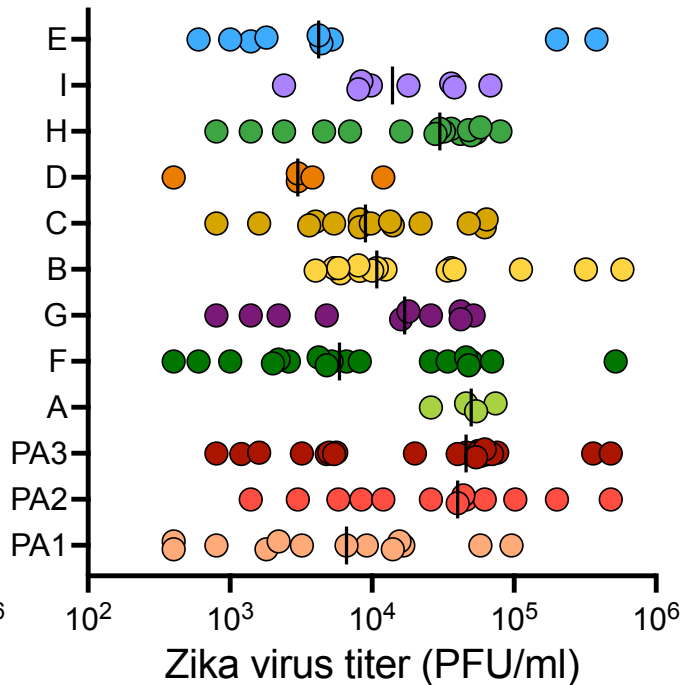**c**

Titers  
(Day 14 - Not disseminated)

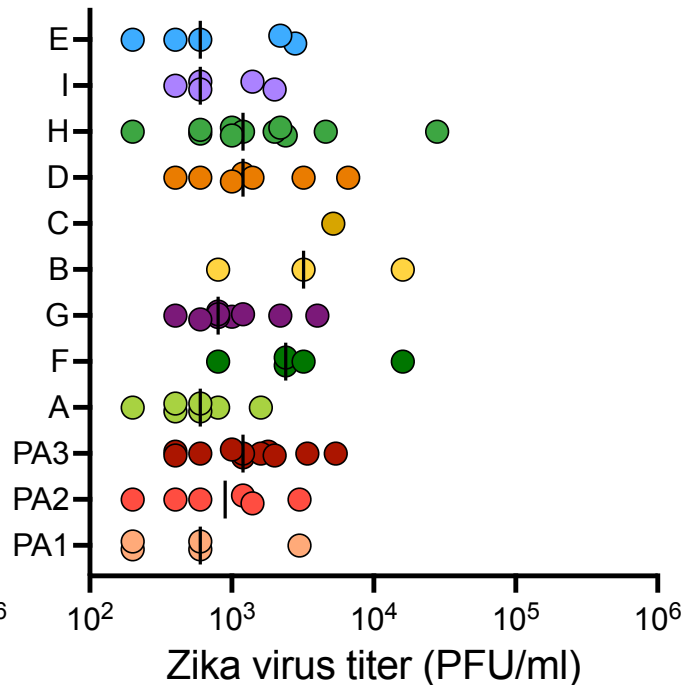

Supplement: S3 Fig — We determined titers of the 12 Zika virus clades in Ae. aegypti (Poza Rica, Mexico) mosquito bodies. We determined Zika titers for (a) engorged mosquitoes at day 0, as well as (b) fully disseminated and (c) infected (but not disseminated) mosquitoes at day 14. Dots represent titers for individual mosquito bodies, and bars indicate the median. Letters indicate significance tested with a Kruskal-Wallis test, followed by a post-hoc Dunn’s test adjusted with false discovery rate (FDR) correction for multiple comparisons. (PDF) [file pntd.0011055.s003.pdf]

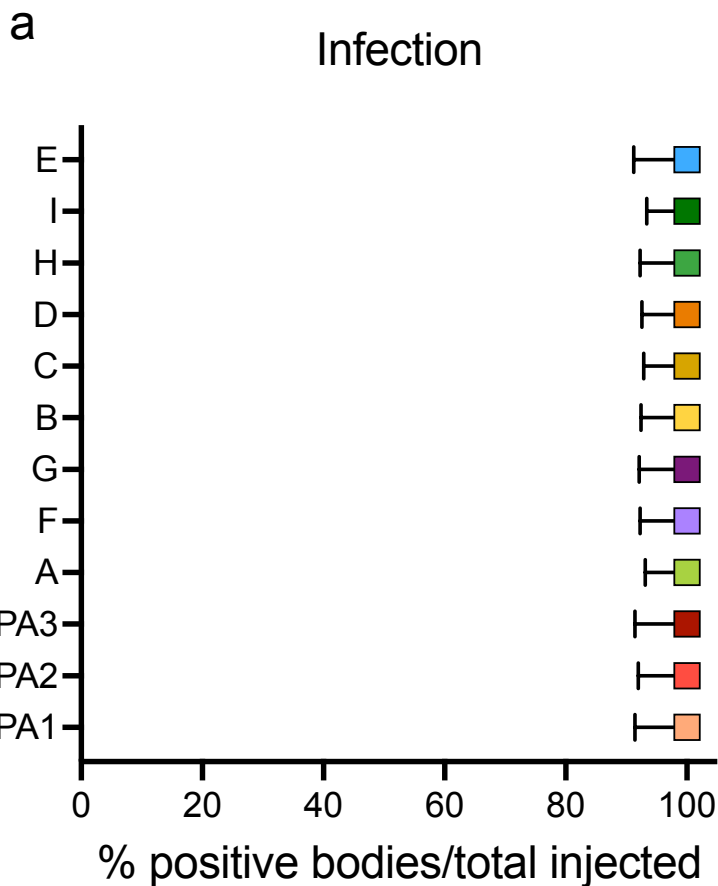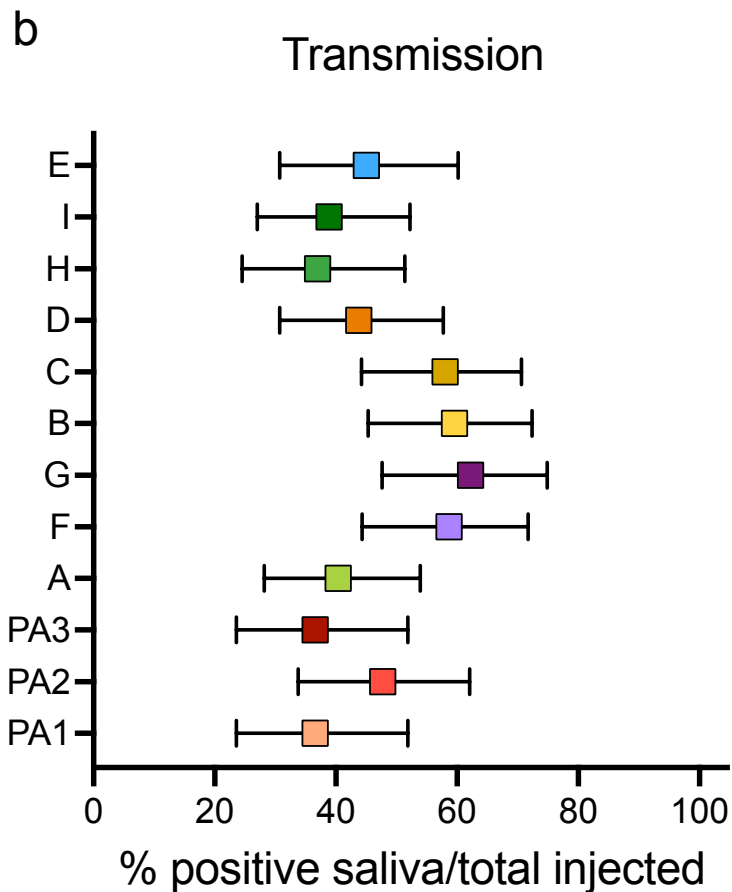

Supplement: S4 Fig — We injected Ae. aegypti (Poza Rica) females with one of the twelve clades. After seven days incubation, (a) all females were confirmed to be infected (Zika virus-positive body), and (b) percentage of females with Zika virus-positive saliva out of the total number of infected females per clade (transmission) were similar among clades. Squares represent the rates expressed as percentages and error bars depict the 95% confidence intervals. We used a Fisher’s exact test for independence to analyze the data, but no significant differences were found. (PDF) [file pntd.0011055.s004.pdf]
