# Supplementary material for: Genomic and phenotypic analyses suggest moderate fitness differences among Zika virus lineages
Source: PLoS Negl Trop Dis. 2023 Feb 8;17(2):e0011055. doi: 10.1371/journal.pntd.0011055 (PMC9907835; doi:10.1371/journal.pntd.0011055)
Supplement: S1 Table — List of lineage-defining mutations and their positions in the Zika virus genome (Isolate Paraiba_01). (PDF) [file pntd.0011055.s005.pdf]

Extended Data Table 1 | Lineage-defining nonsynonymous mutations

Clade	Gene	Gene location	Ancestral amino acid	Mutant amino acid	Mutant genome site	Mutant nucleotide
A	NS1	349*	M	V	3534	G
B	No changes needed, Paraiba_01 is this genotype					
C	NS5	322	I	V	8631	G
	NS5	878	D	E	10301	G
D	NS5	322	I	V	8631	G
	NS5	808	T	I	10090	T
	NS5	878	D	E	10301	G
E	NS1	349*	M	V	3534	G
	NS1	100	G	A	2788	C
	NS3	572	M	L	6327	T
	NS5	525	R	C	9240	T
F	NS1	349*	M	V	3534	G
	Capsid	107	D	E	428	A
G	NS1	349*	M	V	3534	G
	Capsid	107	D	E	428	A
	NS1	324	R	W	3459	T
	NS5	833	T	A	10164	G
H	C	80	I	T	346	C
	NS1	349*	M	V	3534	G
I	Capsid	80	I	T	345	T
	NS1	349*	M	V	3534	G
	NS5	267	V	A	8467	C
J	NS1	349*	M	V	3534	G
	NS1	100	G	A	2788	C
	NS3	572	M	L	6327	T
	NS3	40	V	I	4731	A
	NS5	525	R	C	9240T	T
PA1	NS1	349*	M	V	3534	G
	NS5	114*	M	V	8007	G
PA2	NS1	349*	M	V	3534	G
	prM	17*	S	N	523	A
	NS3	584*	Y	H	6363	C
	NS5	114*	M	V	8007	G
PA3	NS1	349*	M	V	3534	G
	NS5	114*	M	V	8007	G
	prM	17*	S	N	523	A
	NS3	584*	Y	H	6363	C
	NS1	194	V	A	3070	C

*Paraiba_01 infectious clone was reverted to its ancestral amino acid state.
