# Supplementary material for: Genomic and phenotypic analyses suggest moderate fitness differences among Zika virus lineages
Source: PLoS Negl Trop Dis. 2023 Feb 8;17(2):e0011055. doi: 10.1371/journal.pntd.0011055 (PMC9907835; doi:10.1371/journal.pntd.0011055)
Supplement: S2 Table — List of cells that were used to test replicative fitness of infectious Zika virus clones. (PDF) [file pntd.0011055.s006.pdf]

Table 1

Extended Data Table 2 | Experimental Cell types

Cell type	Organism	Tissue type	Description	Prior Zika virus studies
HDF	Human	Fibroblast	Human Dermal fibroblasts	Hamel et al.
HVMF	Human	Fibroblast	Human villous mesenchymal fibroblasts	El Costa et al.
RPE	Human	Epithelial	Retinal pigment epithelial cells	Manangeeswaran et al.
NPC	Human	Neural	Neural progenitor cells	Li et al.
Vero	African green monkey	Epithelial	Kidney epithelial cells	Vicenti et al.
MRC5	Human	Fibroblast	Fibroblasts derived from lung tissue	Vicenti et al.
A549	Human	Epithelial	Adenocarcinomic alveolar basal epithelial cells	Vicenti et al.
Huh7	Human	Epithelial	Hepatocyte-derived carcinoma cell line	Vicenti et al.
SHSY5Y*	Human	Nueral	Neuroblast derived from bone-marrow cell line	Luplertlop et al.
Aag2	*Ae. aegypti*	Mixed	Derived from homogenised embryos	Weger-Lucareli et al., Chouin-Carneiro et al
U4.4	*Ae. albopictus*	Unknown	Derived from neonate larva	Chouin-Carneiro et al
Hsu	*Cx. quinquefaciatus*	Unknown	Derived from ovarian tissue	Guo et al., Weger-Lucareli et al.

*SHSY5Y infections yielded detectable virus during only 1 timepoint and are not included in figure 2.
