# Supplementary material for: Genomic and phenotypic analyses suggest moderate fitness differences among Zika virus lineages
Source: PLoS Negl Trop Dis. 2023 Feb 8;17(2):e0011055. doi: 10.1371/journal.pntd.0011055 (PMC9907835; doi:10.1371/journal.pntd.0011055)
Supplement: S3 Table — Number of live Ae. aegypti mosquitoes included in this study. (PDF) [file pntd.0011055.s007.pdf]

Extended Data Table 3 | Mosquito blood meal Zika virus infections

Zika virus clade	Total number of tested Ae. aegypti mosquitoes	# of positive bodies(infection)	# of positive legs/wings (dissemination)	# of positive saliva (transmission)
PA1	48	18	13	0
PA2	49	19	13	2
PA3	50	32	21	2
A	49	11	4	0
B	41	18	15	2
C	56	15	14	0
D	43	12	5	0
E	59	14	9	0
F	47	24	19	1
G	*41*	19	10	0
H	*59*	28	17	2
I	*48*	13	8	1
